# Organised sports participation and adiposity among a cohort of adolescents over a two year period

**DOI:** 10.1371/journal.pone.0206500

**Published:** 2018-12-05

**Authors:** Stewart A. Vella, Dylan P. Cliff

**Affiliations:** 1 Early Start, Faculty of Social Sciences, Northfields Avenue, University of Wollongong, Wollongong, Australia; 2 School of Psychology, Faculty of Social Sciences, Northfields Avenue, University of Wollongong, Wollongong, Australia; 3 School of Education, Faculty of Social Sciences, Northfields Avenue, University of Wollongong, Wollongong, Australia; CUNY, UNITED STATES

## Abstract

**Background:**

Overweight and obesity among young people is alarmingly high. While hundreds of millions of children participate in organised sports worldwide, it is currently unknown whether time spent in organised sports is associated with levels of adiposity among young people. This study aimed to investigate bidirectional associations between participation in organised sports and adiposity over a two year period.

**Method:**

Data were drawn from the Longitudinal Study of Australian Children. In total, 4033 participants (51% male) reported time spent in organised sports and had their body mass index, body fat percentage, and waist circumference measured at age 12, and again two years later. A cross-lagged panel model was used to examine bidirectional relationships over time, as well as interaction effects.

**Results:**

Total sport participation at age 12 was not associated with subsequent BMI-z scores (*β* = 0.01 [95% CI, -0.02, 0.04]), body fat (*β* = 0.01 [95% CI, -0.02, 0.03]), or waist circumference (*β* = -0.01 [95% CI, -0.05, 0.02]). Similarly, measure of adiposity at age 12 were not associated with subsequent sports participation (BMI-*z* score: *β* = -0.01 [95% CI, -0.02, 0.04]; body fat percentage: *β* = -0.02 [95% CI, -0.05, 0.02]; waist circumference: *β* = -0.01 [95% CI, -0.01, 0.03]). There were no differences in the strength or direction of the relationships by type of sport or by sex (*p* < .05).

**Conclusion:**

Policy and programmatic changes may be needed before organised youth sports are considered a preventative strategy for overweight and obesity. However, a more nuanced understanding of why organised youth sports are not associated with adiposity is needed before evidence-based changes can be made.

## 1. Introduction

Following increases over the last four decades, the prevalence of overweight and obesity among children and adolescents may have stabilised at rates of between 22% and 40% worldwide [1, 2] . Overweight and obesity during childhood and adolescence adversely affects almost every bodily system, and children and adolescents with overweight or obesity are more likely to experience increased rates of cardiovascular diseases and the metabolic syndrome, adverse psychosocial complications, and increased adult mortality [1]. Evidence-based and effective programs and policies are required to stem the negative health effects of high rates of overweight and obesity among children and adolescents worldwide [2]. Programs such as behavioural lifestyle interventions have the potential to lead to clinically meaningful reductions in levels of overweight and obesity [3], while policies to change the food and built environment as well as greater funding for population level prevention programmes are purported as key ingredients to preventing and controlling childhood obesity [4].

One of the most popular contexts for child and adolescent health promotion worldwide is organised sports [5]. It is argued that sport has a critical role to play in preventing the rising rates of morbidity and mortality that stem from non-communicable diseases including overweight and obesity, and that this is especially so among children and adolescents [6]. Worldwide, sport is the one of the most popular contexts for physical activity among young people, with over 40% of children and adolescents participating in sport across most western and non-western countries [5]. However, while sports participation is associated with greater levels of physical activity [7], two successive systematic reviews have demonstrated that the relationship between sports participation during childhood and adolescence and indicators of overweight or obesity such as Body Mass Index (BMI) or waist circumference is inconclusive [7, 8].

It is currently unclear why sports participation may not be associated with overweight or obesity among young people. One reason may be that cross-sectional studies upon which this conclusion is based have been unable to discern directionality in the relationship–is sports participation the consequence or cause of reduced adiposity? This is important because distinct relationships may exist between the two variables over time [8]. For example, if sports participation is equally as popular among overweight and obese children as it is among children of a healthy weight, no clear association between sports participation and weight status would materialise. This remains true even if sports participation is causally related to reduced adiposity over time. In support of potential bidrectionality in the relationship, Cairney and Veldhuizen [9] have recently demonstrated that, among almost 2000 children aged 9–10 years, there are weak bidirectional associations between participation in organised sports and BMI over a five year period.

Further methodological limitations may also contribute to the lack of clear understanding of the relationship between sports participation and adiposity. Overreliance on BMI as an indicator of adiposity could inflate the risk of systematic type II errors because it is potentially confounded by increased muscle mass among sport participants. Alternately, use of binary categorical variables (i.e., sport participants verse non-participants) precludes consideration of the duration of participation, and instead use greater participation in the number of teams as a proxy for greater duration. For example, participation on two sporting teams is associated with meaningful decreases in the risk for overweight and obesity among children [10]. The study conducted by Cairney and Veldhuizen [9] offers preliminary evidence of bidirectional relationships between organised sports participation and adiposity over time, but is potentially confounded by the sole use of BMI as a measure of adiposity, and the use of categorical proxy measures of sports participation. The authors have noted that measurement issues may have influenced the results and recommended that measures of duration, as well as more sensitive measures of adiposity should be included in future research.

The purpose of this study is to replicate and build upon recent evidence provided by Cairney and Veldhuizen [9] to examine the relationship between participation in organised sports and measures of adiposity among young people over a 2-year period. More specifically, this study uses a continuous measure of sports participation and three distinct indicators of adiposity: BMI; waist circumference; and, body fat percentage. Further, this study undertook exploratory analyses to examine whether relationships differ by sex, or type of sport. Hundreds of millions of children participate in organised sports worldwide [5] and a more thorough and nuanced understanding of potential causal influences (if any) could lead to more effective sports programs and policies to combat rising levels of pediatric obesity. In line with recent evidence [9], we hypothesised that weak bidirectional associations between participation in organised sports and measures of adiposity would be evident over the two-year period.

## 2. Method

### 2.1 Study design and participants

All data were drawn from Waves 5 and 6 of the Longitudinal Study of Australian Children (LSAC). LSAC is a biennial study of the social, environmental, and economic influences on the health and development of Australian children. Participants were selected at random from the most comprehensive database of the Australian population (Medicare database) in 2004, and Waves 5 and 6 were collected in 2012 and 2014. Data used in this study were collected from the Kindergarten (K) cohort of LSAC where children were aged 4–5 years at Wave 1 and 12–13 years at Wave 5. Data used in this study were collected by trained professionals using face-to-face interviews with study participants and their primary parent (the child’s mother in 96% of cases). At Wave 1, the response rate among all invited participants for the K-Cohort was 50%, resulting in an initial sample size of 4983.

**Fig 1:**
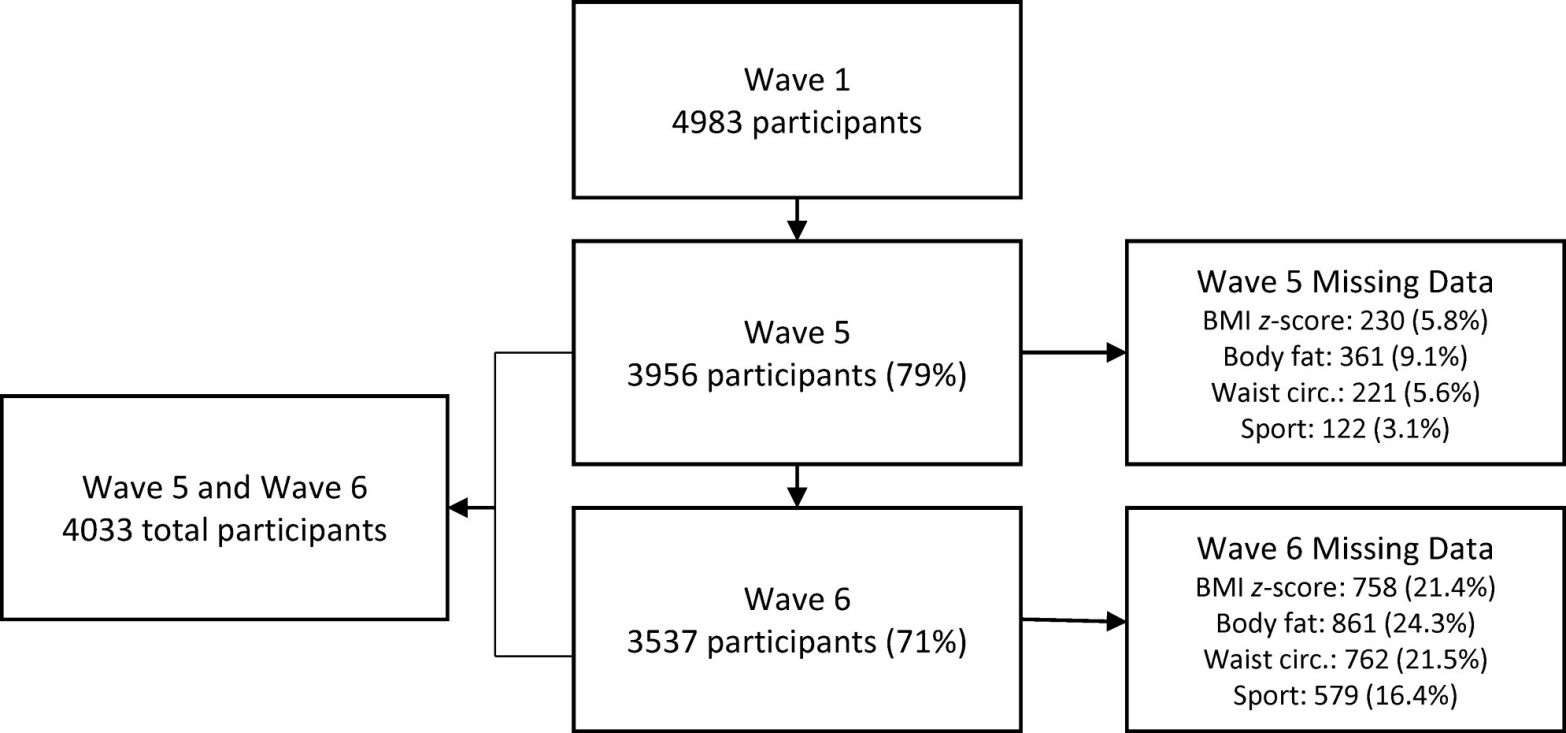
Flow chart.

This study utilised data obtained during Wave 5 and Wave 6 as they are the only waves where a measure of time spent in extracurricular organised sport was included. Sport data were reported by the primary parent of the participant and indicators of adiposity were measured by the trained professionals. Participants were included if they had complete sports participation data in either Wave 5 or 6. In total, 4033 participants were included and this represents an attrition rate of 19% from Wave 1. A detailed description of the study and data collection methodology has been reported elsewhere [11]. A flow chart of participants in the current study is given in Fig 1. Ethical approval for the LSAC study was granted by the University of Melbourne’s Human Research Ethics Committee. Parents provided written informed consent prior to participation.

### 2.2 Measures

#### 2.2.1 Sport participation

Average weekly time spent in team and individual sports was calculated from six parental reported items. First, two items assessed regular participation in either team or individual sports outside of school hours. For extracurricular team sports, parents were asked “In the last week, has (your) child participated in team sport (e.g. football, cricket or netball)?” For extracurricular individual sports, parents were asked “In the last week, has (your) child regularly participated in individual sport (e.g. tennis, karate or gymnastics)”? Second, if children participated in extracurricular sports, parents were asked to report the average number of days that the child participated in team and/or individual sports each week, as well as the average number of hours per day. Hours per day was categorised as: up to 1 hour per day; more than 1 but less than 2 hours per day; more than 2 hours per day. To calculate average weekly time in organised sports we multiplied the number of days of participation by the median value for number of hours per day. For the category ‘up to one hour’ we multiplied by 0.5 hours. For the category ‘more than 1 but less than 2 hours’ we multiplied by 1.5 hours. Finally, for the category of ‘more than 2 hours’ we multiplied by 2.5 hours. Parents reporting that their child did not participate in sport were scored as 0 hours participation per week. This measure of sports participation has demonstrated associations with a range of demographic, socioeconomic, and environmental factors known to be associated with health and health behaviours [12]. Furthermore, the resulting variable is the most accurate, understandable and meaningful measure of sports participation available (i.e., in hours per week, combining both frequency *and* duration), is consistent with the gold standard in measurement of sports participation [13], and the data suggest that the measure is valid (data are consistent with the distribution observed in population studies) [14].

#### 2.2.2 Adiposity

Trained professionals measured participants’ height, weight and body fat. Weight and body fat percentage were measured using digital body fat scales (Tanita Australia, Kewdale, Australia). Weight was recorded to the nearest 0.05 kg. Height was measured using the average of two measurements taken by portable laser stadiometer (Invicta, Leicester, United Kingdom). If measurements differed by more than 0.5 cm a third measure was taken and the average of the two closest measures was used. BMI was calculated by dividing the participant’s weight in kg by the square of their height in metres. BMI z-score were used in the current study to account for differences in BMI by sex, and were calculated based on the 2000 US Centers for Disease Control growth charts [15]. Waist circumference was measured using the average of two measures taken by placing a non-stretch dressmakers tape horizontally over the navel. If measurements differed by more than 0.5cm a third measure was taken and the average of the two closest measures was used.

#### 2.2.3 Covariates

All covariates were assessed at wave 5 when children were aged 12 years, and included child sex, neighbourhood socioeconomic position (SEP), language spoken at home, dietary behaviours, pubertal status, and type of sport. The primary parent reported the child’s sex, home postcode, and language spoken at home. Neighbourhood SEP was determined according to the Socio-Economic Indexes for Areas Index of Relative Socio-Economic Disadvantage [16] using the child’s home postcode. Language spoken at home was self-reported and was categorised as English or non-English. Obesity-related dietary behaviours included two variables assessing participants’ daily serves of high fat foods, and daily serves of sugary drinks. Daily serves of high fat foods was calculated using the sum of four items assessing serves of: meat pie, hamburger, hot dog, sausage, or sausage rolls; hot chips or French fries; potato chips or savoury snacks; and, biscuits, doughnuts, cakes, or chocolate. Daily serves of sugary drinks was assessed using the number of serves of soft drink or cordial. Each item used the stem “Thinking about yesterday, how often did you have…” and was coded as ‘not at all’, ‘once’, ‘twice’ or ‘more than twice’. Pubertal status was measured using facial hair growth for males and breast growth for females. Answers were reported by the primary parent and were categorised as ‘has not started yet’, ‘has barely started’, ‘has definitely started’, and ‘seems complete’. Type of sport was assessed using the first two parent-report items regarding their child’s regular participation in extracurricular team and individual sports as described above. Data were categorised as: No Sport; Individual Sport Only; Team Sport Only; or, Team and Individual Sport.

### 2.3 Statistical analyses

**Fig 2:**
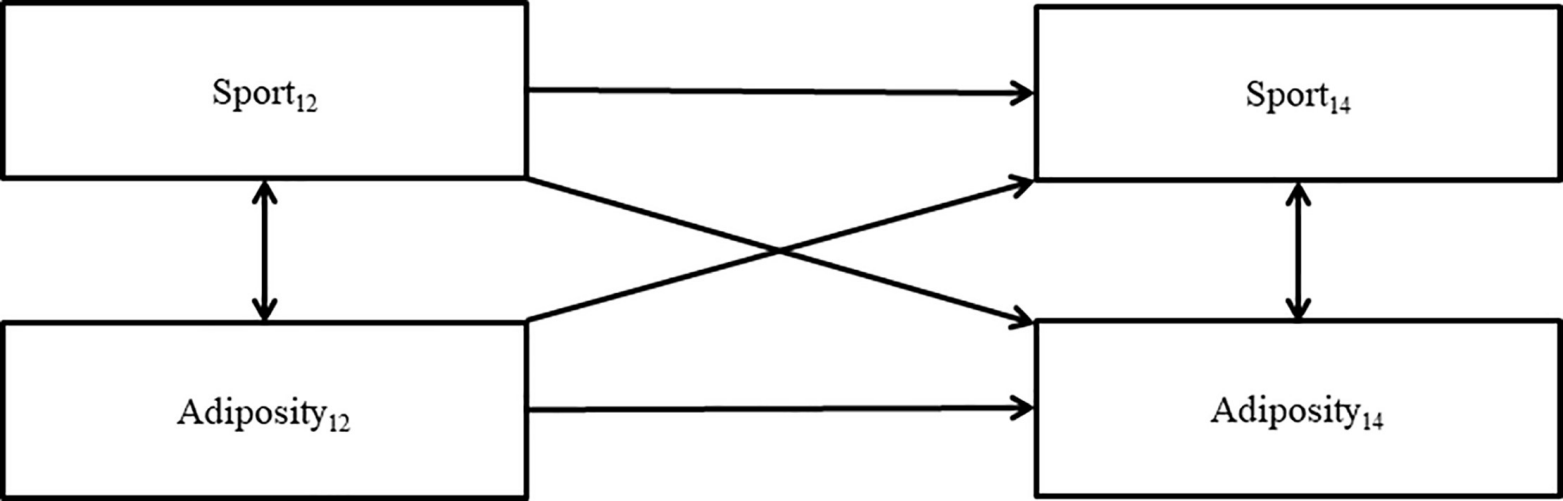
Cross-lagged panel model.

To test potential bi-directional relationships between sports participation and adiposity we used cross-lagged panel models analysed in M*plus* version 6.11 [17]. Cross-lagged panel models enable the simultaneous examination of reciprocal relationships between two variables over time by testing stability paths (e.g., adiposity at age 12 → adiposity at age 14), concurrent paths (e.g., sport participation at age 12 → adiposity at age 12), and cross-lagged paths (e.g., sport participation at age 12 → adiposity at age 14; adiposity at age 12 → sport participation at age 14). Three distinct models were tested for total time spent in sport and the three measures of adiposity (BMI, body fat, waist circumference). To account for any potential response bias, and to account for relevant confounding variables, each model controlled for all covariates. Missing data were estimated in Mplus using the full information maximum likelihood method. Fig 2 provides a graphical representation of the cross-lagged panel model.

The multiple group function in M*plus* was used to test potential moderating effects of sport type or sex. This is done by constraining the lagged paths to be equal across groups [17] and then subsequently re-testing the model with one lagged path unconstrained. The chi-square difference relative to the fully constrained model was derived. A statistically significant difference between the chi-square statistics indicated that the unconstrained lagged path differed significantly between groups and it could be concluded that an interaction effect was present. This was sequentially repeated for each lagged path. Significance was set a *p* < .05.

## 3. Results

### 3.1 Participants

Across the two waves of data there were a total of 4033 eligible adolescents (51% male and 49% female). Average age at Time 1 was 12.41 years (*SD* = 0.49 years). At Time 1 1510 participants (39%) did not participate in sport, 526 (13%) participated in individual sports, 1337 (34%) participated in team sports, and 538 (14%) participated in both individual and team sports. Mean time spent in organised sport and mean scores for measures of adiposity by sex and sport participation are provided in Table 1. As expected, total sports participation at age 12 and at age 14 were positively skewed with an expected tendency toward zero inflation (the mode for each age was zero participation each week). The median value for sports participation 1.5 hours at age 12, and 0.5 hours at age 14. The distribution for sports participation data at both ages 12 and 14 years was consistent with the expected distributions of sports participation during adolescence. Given that data were distributed as expected and that the use of robust maximum likelihood estimation using the Mplus program allows for non-normally distributed data, the data were not treated as problematic [17].

**Table 1:** Mean sport participation and measures of adiposity by sex, sport type and weight status.

	N (%)	Mean Time Spent in Sport Participation [hrs.wk^-1^] (SD)						Mean Adiposity (SD)					
		Time 1 (12 years)			Time 2 (14 years)			Time 1 (12 years)			Time 2 (14 years)		
		Individual	Team	Total	Individual	Team	Total	BMI	Body Fat	Waist	BMI	Body Fat	Waist
Total Sample	4033	0.81 (2.05)	1.77 (2.64)	2.58 (3.36)	0.76 (2.13)	1.68 (2.76)	2.45 (3.53)	20.48 (3.89)	21.23 (9.56)	71.88 (10.30)	21.94 (4.14)	21.00 (9.96)	75.11 (10.18)
Males	2020 (51)	0.77 (1.93)	2.17 (2.90)	2.94 (3.51)	0.77 (2.10)	2.01 (2.99)	2.78 (3.65)	20.24 (3.84)	17.91 (9.02)	72.82 (10.77)	21.61 (4.15)	16.31 (8.67)	76.92 (10.70)
Females	1936 (49)	0.84 (2.18)	1.36 (2.26)	2.20 (3.14)	0.77 (2.18)	1.35 (2.46)	2.11 (3.39)	20.73 (3.93)	24.74 (8.83)	70.91 (9.70)	22.29 (4.13)	26.02 (8.68)	73.15 (9.25)
Sport at Age 12													
Nil	1510 (39)	-	-	-	0.40 (1.62)	0.70 (1.75)	1.11 (2.41)	20.82 (4.28)	22.61 (10.15)	73.02 (11.37)	22.23 (4.74)	21.92 (10.54)	75.56 (11.30)
Individual	526 (13)	3.30 (3.32)	-	3.30 (3.32)	1.73 (2.98)	0.81 (1.77)	2.54 (3.31)	20.00 (3.58)	20.44 (8.87)	70.13 (9.64)	21.53 (3.83)	20.53 (9.50)	73.61 (9.71)
Team	1337 (34)	-	3.72 (2.69)	3.72 (2.69)	0.49 (1.65)	2.53 (3.11)	3.01 (3.59)	20.57 (3.77)	20.80 (9.36)	72.17 (9.82)	21.99 (3.80)	20.46 (9.65)	75.49 (9.58)
Both	538 (14)	2.63 (2.68)	3.63 (2.77)	6.26 (4.13)	1.43 (2.74)	3.04 (3.47)	4.48 (4.47)	19.75 (3.08)	19.26 (8.44)	69.75 (8.20)	21.39 (3.35)	20.01 (9.08)	74.01 (8.45)
Weight Status													
Underweight	260 (7)	0.95 (2.43)	1.22 (2.04)	2.17 (3.17)	0.69 (1.64)	1.41 (2.59)	2.10 (3.11)	15.10 (0.80)	12.99 (6.18)	61.10 (5.25)	17.27 (2.22)	13.88 (7.73)	65.59 (5.83)
Healthy	2549 (67)	0.92 (2.23)	1.92 (2.77)	2.84 (3.55)	0.81 (2.26)	1.77 (2.78)	2.58 (3.57)	18.99 (1.65)	18.62 (7.37)	68.16 (5.49)	20.62 (2.22)	19.12 (8.32)	71.98 (6.06)
Overweight	744 (18)	0.53 (1.49)	1.70 (2.46)	2.23 (2.90)	0.69 (1.91)	1.68 (2.80)	2.37 (2.58)	24.21 (1.45)	27.70 (7.83)	80.40 (7.20)	25.44 (3.24)	26.73 (10.07)	82.75 (8.99)
Obesity	250 (6)	0.46 (1.31)	1.30 (2.26)	1.76 (2.59)	0.62 (1.63)	1.13 (2.54)	1.75 (3.17)	30.14 (2.79)	36.61 (9.98)	94.59 (10.21)	31.62 (4.30)	32.68 (12.18)	97.78 (11.50)

### 3.2 Body mass index

No relationship was evident between sports participation and BMI-*z* scores in either direction over time (Table 2). Total sport participation at age 12 was not associated with BMI-*z* scores at age 14 (*β* = 0.01 [95% CI, -0.02, 0.04]). Similarly, BMI-*z* scores at age 12 was not associated with sport participation at age 14 (*β* = -0.01 [95% CI, -0.02, 0.04]). There were no significant differences in either relationship by type of sport (BMI-*z* → Sport: χ^2^ = 1.98, *p* = .372; Sport → BMI-*z*: χ^2^ = 0.65, *p* = .723) or by sex (BMI-*z* → Sport: χ^2^ = 2.10, *p* = .147; Sport → BMI-*z*: χ^2^ = 0.02, *p* = .888).

**Table 2:** Path coefficients for cross-lagged panel models.

Adiposity Measure	Concurrent Paths		Stability Paths		Cross-Lagged Paths	
	T1 (12 years)	T2 (14 years)	Time in Sport	Adiposity	Sport_12_ → Adiposity_14_	Adiposity_12_ → Sport_14_
Unstandardised Results [*b* (95%CI)]						
BMI-*z*	-0.15(-0.26, -0.04)**	-0.03 (-0.13, 0.06)	0.44 (0.41, 0.47)**	0.78 (0.75, 0.81)**	0.01 (-0.01, 0.01)	0.03 (-0.08, 0.13)
Body Fat	-4.36 (-5.40, -5.75)**	-1.21 (-2.05, -0.36)**	0.44 (0.41, 0.47)**	0.48 (0.45, 0.51)**	0.02 (-0.07, 0.10)	0.00 (-0.02, 0.01)
Waist	-3.20 (-4.64, -2.10)**	-0.80 (-1.70, -0.11)*	0.44 (0.41, 0.47)**	0.80 (-1.48, -0.11)**	0.01 (-0.04, 0.09)	-0.01 (-0.02, 0.00)
Standardised Results [*β* (95%CI)]						
BMI-*z*	-.04 (-0.08, -0.01)**	-0.01 (-0.05, 0.03)	0.42 (0.39, 0.45)**	0.70 (0.68, 0.72)**	0.02 (-0.01, 0.04)	0.01 (-0.02, 0.04)
Body Fat	-0.14 (-0.17, -0.10)**	-0.05 (-0.09, -0.02)**	0.42 (0.39, 0.45)**	0.46 (0.43, 0.48)**	0.01 (-0.02, 0.03)	-0.02 (-0.05, 0.02)
Waist	-0.09 (-0.12, -0.06)**	-0.04 (-0.08, -0.01)*	0.42 (0.39, 0.45)**	0.79 (0.77, 0.80)**	0.01 (-0.01, 0.03)	-0.01 (-0.05, 0.02)

Note.

** *p* < .01

* *p* < .05

Concurrent paths represent the cross-sectional association between sport participation and adiposity at a given time point; Stability paths represent the association between sports participation at Time 1 with sports participation at time 2, or adiposity at Time 1 with adiposity at Time 2.

### 3.3 Body fat

No relationships were evident between body fat and time spent in organised sport over time (Table 2). Total sport participation at age 12 was not associated with body fat two years later (*β* = 0.01 [95% CI, -0.02, 0.03]). Body fat at age 12 was not associated with total sport participation two years later (*β* = -0.02 [95% CI, -0.05, 0.02]). There were no significant differences in the strength of either relationship by type of sport (Body Fat → Sport: χ^2^ = 1.02, *p* = .600; Sport → Body Fat: χ^2^ = 0.16, *p* = .923) or by sex (Body Fat → Sport: χ^2^ = 0.01, *p* = .920; Sport → Body Fat: χ^2^ = 0.36, *p* = .549).

### 3.4 Waist circumference

Waist circumference at age 12 did not predict total sport participation at age 14 (*β* = -0.01 [95% CI, -0.05, 0.02]), and total sport participation at age 12 did not predict waist circumference at age 14 (*β* = -0.01 [95% CI, -0.01, 0.03]). There were no significant differences by type of sport (Waist → Sport: χ^2^ = 0.10, *p* = .951; Sport → Waist: χ^2^ = 2.08, *p* = .353) or by sex (Waist → Sport: χ^2^ = 2.82, *p* = .093; Sport → Waist: χ^2^ = 0.01, *p* = .920).

## Discussion

This study examined potential bidirectional relationships between sports participation and adiposity over time using three distinct indicators of adiposity: BMI-*z*; waist circumference; and, body fat percentage. No relationships were evident for any indicator of adiposity in either direction over the two-year period. Furthermore, the strength or direction of the relationships did not differ by the type of sport played or by child sex. This suggests that, despite promising results that participation in well-controlled, sport-based interventions can reduce adiposity among children and adolescents [18–20], participation in extracurricular organised sports programs as they are currently implemented in Australia has no relationship with young people’s levels of adiposity between 12 and 14 years-of-age.

Although the effect sizes demonstrated in the study by Cairney and Veldhuizen [9] are small, the current results differ from that study in a meaningful way where relationships were evident in both directions over time. Perhaps the most important difference is that the previous study used lagged associations over a five year period, whereas the current study used only a two-year follow-up period. It is possible that the relationships between participation in organised sports and adiposity gets stronger over time. Alternatively, relationships may be slightly stronger when initial outcomes are measured at age 9–10 years, rather than 12–13 years. As such, it may be that earlier sports participation and adiposity are more predictive of subsequent changes than later measures. Nonetheless, when taken together, both studies provide evidence that the link between sports participation and adiposity over time during adolescence is not strong. In fact, the associations may be weak at best, or non-existent at worst.

The assumption that sports participation will lead to health benefits including lower rates of overweight and obesity is pervasive [6]. However, outside of well-controlled, sports-based intervention programs, there is very little evidence to suggest that sports participation is strongly associated with levels of adiposity [8]. Notwithstanding potential confounding variables that have not been controlled for, this study demonstrates that, even after allowing for the possibility that the universal popularity of sports may be ameliorating observed associations between sports participation and adiposity, greater amounts of sports participation were not associated with decreases in any measure of adiposity over time. This suggests that the current design, implementation, and context of extracurricular organised sports in Australia, while popular, are not conducive to the prevention of overweight and obesity in children and youth. It is currently unclear why this is the case. However, evidence suggests that greater caloric intake among youth sports participants [7] mitigates the benefits of greater levels of physical activity [7, 8]. Alternatively, large amounts of practice time in organised youth sports are spent in sedentary or light activities, with only one-third to half of the time spent in moderate to vigorous physical activity [21, 22]. It may also be the case that one’s patterns of sport participation over time period of greater than 2 years are also important. Tracking individual patterns of participation in organised youth sports, in particular by weight status, may generate additional insights regarding the relationships between sport participation and adiposity over time.

Interestingly, no relationship was evident between sports participation and adiposity among those who participated in more than one type of sport. This is in contrast to evidence of a relationship provided by studies which used categorical measures of sports participation [10, 23, 24]. As such, it remains unclear whether or how the breadth of sports experience (e.g., number or type of sports participated in) or the amount of sports participation (e.g., hours per week) influence health-related outcomes including adiposity. In particular, the effectiveness of organised youth sports as they are currently implemented, as opposed to the efficacy of well-controlled sporting programs, should be the focus of future research to discern any potential causal influence of breadth and duration of sports participation on health including reductions in levels of adiposity.

No relationship was evident between levels of adiposity at age 12 years and subsequent sports participation. It appears that overweight and obese children are participating in organised extracurricular sports for the same duration as those of a healthy weight. This is important because reduction in sports participation appears prevalent during adolescence [25], and overweight/obesity is associated with declines in subsequent physical activity over this time [1]. This finding therefore reinforces, and potentially perpetuates, the notion that sports can be used as a universal, engaging, and motivating vehicle for the accumulation of health benefits [6], particularly among overweight and obese young people. For public health benefits, more must be done to improve the design and delivery of organised youth sports to target improvements in health, including changes at both the policy and programmatic levels. The universal popularity of sports lays the platform for the materialisation of public health benefits given effective changes.

That relationships did not differ by type of sport or by sex suggests that nature of the sport or the participant may not be of meaningful importance in the relationship between the duration of participation and adiposity. In fact, there may be greater differences between any given two sports than systematic differences between sports categorised as ‘individual’ or ‘team’ sport. For example, the intensity and duration of physical activity inherent in swimming is potentially very different to that inherent in golf, despite both being categorised as individual sports. Furthermore, unhealthy food options are likely to be equally available across all types of sports.

Strengths of this study include the large sample and the prospective nature of the study design that enable associations to be studied over time. Furthermore, the use of a continuous variable assessing duration of sports participation per week and the use of three distinct measures of adiposity are also strengths. However, there are also limitations that need to be considered. First, this study is unable to account for the amount of physical activity undertaken during sports participation, which could be an important mediating variables in relationships between sports participation and adiposity over time. Future research should include objective measures of physical activity. Second, sports are a context for health benefits, rather than a health behaviour, and we are unable to account for contextual variables such as exposure to unhealthy food advertising within sports. Third, the two-year timeframe allowed us to analyse whether reciprocal relationships between sports participation and adiposity unfolded over time, but it’s possible that these relationships may differ over shorter or longer periods of time. Further, this study utilised a parent-reported measure of time spent in extracurricular sports that was derived from two distinct items measuring frequency of participation in days per week and average duration of participation in hours per day. This approach is closely related to the measures considered as the gold standard in measuring sports participation including the Adolescent Physical Activity Recall Questionnaire [13] which measure frequency and duration separately to provide the most reliable measure of time spent in organised sports. Notably however, the use of an arbitrary median value for duration of participation means that the resultant measure can only be considered a proxy measure of time spent in sports. Lastly, while waist circumference is a valid anthropometric proximal measure of abdominal obesity, waist-to-height ratio may be a more suitable measure among adolescents who are still growing [26].

Randomised controlled trials have shown that sports participation can be used to facilitate health benefits, including decreased levels of adiposity, among overweight and obese children [18–20]. However, organised extracurricular sports programs as they are currently participated in, governed, designed, and implemented had no association with levels of adiposity over time among 12- to 14-year-old Australian children and adolescents. Nonetheless, the popularity of sports supports the potential that they may be used for public health benefits. Policy and programmatic changes may be needed before organised youth sports are considered a preventative strategy for overweight and obesity. However, a more nuanced and detailed understanding of the how and why organised youth sports do not lead to benefits to adiposity is needed before evidence-based changes can be made. Future research should focus on delineating the causal pathways through which sports participation influences adiposity, with a particular focus on the effectiveness of organised youth sport programs as they currently exist. Through this knowledge, changes can be implemented which may lead to meaningful health benefits for the hundreds of millions of children who participate in organised sports worldwide [5].
